# Long-Term Subjective Outcomes of Barbed Reposition Pharyngoplasty for Obstructive Sleep Apnea Syndrome Treatment

**DOI:** 10.3390/ijerph17051542

**Published:** 2020-02-27

**Authors:** Giannicola Iannella, Bianca Vallicelli, Giuseppe Magliulo, Giovanni Cammaroto, Giuseppe Meccariello, Andrea De Vito, Antonio Greco, Stefano Pelucchi, Rossella Sgarzani, Ruggero Massimo Corso, Gloria Napoli, Giulia Bianchi, Salvatore Cocuzza, Antonino Maniaci, Claudio Vicini

**Affiliations:** 1 Department of ‘Organi di Senso’, University “Sapienza”, Viale dell’Università, 33, 00185 Rome, Italy; giuseppe.magliulo@uniroma1.it (G.M.); antonio.greco@uniroma1.it (A.G.); 2 Department of Head-Neck Surgery, Otolaryngology, Head-Neck and Oral Surgery Unit, Morgagni Pierantoni Hospital, Via Carlo Forlanini, 34, 47121 Forlì, Italy; giovanni.cammaroto@hotmail.com (G.C.); drmeccariello@gmail.com (G.M.); dr.andrea.devito@gmail.com (A.D.V.); claudio@claudiovicini.com (C.V.); 3 Department ENT & Audiology, University of Ferrara, Via Savonarola, 9, 44121 Ferrara, Italy; bianca.vallicelli@student.unife.it (B.V.); stefano.pelucchi@unife.it (S.P.); nplglr@unife.it (G.N.); giuwhites91@gmail.com (G.B.); 4 Department of Emergency, Burn Center, Bufalini Hospital, Azienda USL della Romagna, viale Giovanni Ghirotti, 286, 47521 Cesena, Italy; rossellasgarzani@libero.it; 5 Intensive Care Unit, Morgagni-Pierantoni Hospital, AUSL of Romagna, Via Carlo Forlanini, 34, 47121 Forlì, Italy; mcorso@gmail.com; 6 Department of Otolaryngology, University of Catania, Via S. Sofia, 78, 95125 Catania, Italy; s.cocuzza@unict.it (S.C.); tnmaniaci29@gmail.com (A.M.)

**Keywords:** obstructive sleep apnea, pharyngoplasty, barbed reposition pharyngoplasty, questionnaire, obstructive sleep apnea surgery

## Abstract

*Background*: The purpose of this study was to evaluate long-term subjective outcomes of barbed reposition pharyngoplasty for obstructive sleep apnea syndrome (OSAS) treatment using a specific questionnaire, the Palate Postoperative Problem Score (PPOPS). *Methods*: 140 patients who underwent barbed reposition pharyngoplasty (BRP) surgery in the Morgagni Pierantoni Hospital of Forlì, Italy were enrolled in the study. Postoperative outcomes were evaluated in a short- and long-term follow-up using the PPOPS questionnaire. The average period of follow-up was 26 months. All patients received the PPOPS questionnaire by telephone in a period between April and August 2019. *Results*: 51% of patients complained of swallowing problems after surgery. In 91% of cases, the problem cleared up spontaneously. At the time of the interview, only 9% of patients had a residual swallowing difficult. At the time of PPOPS evaluation, rhinolalia was observed in 8% of patients, whereas nose regurgitation was present in 2% of patients. In 20% of patients, the foreign body sensation was present during follow-up. The value of apnea–hypopnea index (AHI) reduced from the preoperative value of 31.5 to the postoperative value of 11.4. *Conclusions*: BRP surgery proved to be an effective technique, appreciated by the majority of patients. Use of the PPOPS questionnaire has demonstrated that the BRP technique seems to ensure efficacy and lower morbidity, with few complications after surgery.

## 1. Introduction

“Obstructive Sleep Apnea (OSA) syndrome is a respiratory sleep disorder characterized by partial or complete recurrent episodes of upper airway collapse, that occurs during the night” [1,2]. OSA manifests with a reduction (hypopnea) or complete cessation (apnea) of airflow in the upper airways [2,3,4].

Although multilevel collapse of the upper airway is not rare during sleep in patients with OSA, the velopharyngeal region is believed to be the most common site of obstruction, and lateral pharyngeal wall collapse was considered a determining factor in velopharyngeal obstruction [5,6,7].

Consequently, palatal surgical techniques have changed throughout recent years from the classic uvulopalatopharyngoplasty (UPPP) to lateral pharyngoplasty techniques [6,7,8].

Of the different lateral pharyngoplasty techniques used, barbed reposition pharyngoplasty (BRP) has recently become one of the most practiced palatal surgery techniques in different countries. BRP was described in a pilot study by Vicini et al. [9] in 2015, as a quick, easy, and effective technique for treating velopharyngeal obstruction in OSA patients. Authors proposed the use of the barbed suture (knotless bidirectional resorbable suture) for obtaining suspension of the palatopharyngeal muscle at the pterygomandibular raphe and expansion of the lateral walls of the oropharynx without tissue resection of the soft palate region [6,9].

The role of BRP was confirmed by Montevecchi et al. [10] in a prospective multicentric study published in 2017 and by Madkikar et al. [11] in a subsequent prospective study. In both studies, BRP proved to be a simple, easy to learn, and safe procedure with promising results in the management of OSA and in single and multilevel surgery.

Pharyngoplasty techniques are often accompanied by complications and consequences. In the short and long term, the most typical problems reported in these patients are dysphagia, rhinolalia, velopharyngeal insufficiency and nasopharyngeal regurgitation, phlegm in throat, foreign body sensation, and velopharyngeal stenosis. Another important aspect to keep in mind is postoperative pain, especially during swallowing [12,13,14]. These complications were often reported for UPPP and laser-assisted uvulopalatoplasty (LAUP) where major palate resections were performed [15,16]. On the other hand, lateral pharyngoplasty techniques would seem to have a lower incidence of these postoperative complications [12,13].

In order to clarify this aspect, Pang et al. [12], in 2019, published a multicenter study about long-time complications of palatal surgery. In this study, 217 patients were involved, to compare complications of new palatal techniques such as BRP with older techniques, such as expansion sphincter pharyngoplasty (ESP), UPPP, uvulopalatal flap (UPF), suspension pharyngoplasties (SP), and relocation pharyngoplasties (RP), performed in six different countries.

The complications recorded in the study group were dry throat (7.8%), foreign body sensation (11.5%), phlegm in throat (10.01%), throat scar feeling and difficulty swallowing (0.5%). Forty BRP patients were included. Only seven patients who underwent BRP had a worse feeling in the throat: foreign body sensation in one case, dry throat in two cases, phlegm in four cases. In all cases, these sensations were present occasionally.

One important aspect that should be considered is that many of the postoperative complications of palatal surgery are related to the patient’s subjective perceptions. Therefore, to accurately evaluate the outcome of surgery it is necessary to explore subjective perception after such procedures.

In order to investigate subjective outcome of palatal surgery, in 2018, Rashwan et al. [17] designed a questionnaire, the Palate Postoperative Problem Score (PPOPS). This questionnaire investigated the patients’ perception and the most common problems arising after surgery in the long and short term. The questions in the questionnaire refer both to the days after surgery and to the time of the interview. In the pilot study, PPOPS was presented to 40 patients with OSA who underwent BRP surgery, in comparison with ESP. The PPOPS questionnaire was defined as a useful tool for the assessment of palatal surgery through a detailed analysis of the patients’ perception of the surgery they had undergone.

The aim of this study was to analyze long-term subjective outcomes in a large group of patients undergoing barbed reposition pharyngoplasty. Subjective perception and postoperative problems referred by patients have been investigated using the PPOPS questionnaire.

## 2. Materials and Methods

### 2.1. Enrollment of the Patients and Inclusion/Exclusion Criteria

The study initially considered 271 OSA patients who underwent BRP surgery between January 2015 and February 2019 at the Otolaryngology Unit of the Morgagni–Pierantoni Hospital in Forlì, Italy. The study included patients who had undergone BRP surgery in a single stage or combined with nasal surgery (septoplasty and/or turbinoplasty). Patients who underwent BRP in multilevel surgery with transoral robotic surgery (TORS) to perform a resection of the tongue base and/or epiglottoplasty were excluded from the study due to the possible bias of TORS in pharyngeal outcomes.

All patients were contacted by phone. Patients who refused the questionnaire or those lost to follow-up were excluded from the study. Patients who underwent BRP for revision of previous surgery were also excluded from the study.

### 2.2. Patient’s Characteristics

One hundred and forty patients were enrolled in the study.

The average age of the study group was 49 years, in a range between 24 and 81 years old.

Patients were divided into three age groups: <40 years, between 40 and 65 years, and >65 years. There were 33 patients aged <40 (24%), 94 aged from 40 to 65 years (67%), and 13 aged >65 years (9%). There was an evident difference in sex incidence of the patients enrolled: 10 (7%) were females and 130 (93%) males.

In accordance with the American Academy of Sleep Medicine (AASM) [18,19], diagnosis and classification of OSA was made on the basis of the apnea–hypopnea index (AHI) obtained from polysomnography study (PSG). Patients were classified as either mild OSA (AHI ≥ 5 and <15), moderate (AHI ≥ 15 and <30), or severe OSA (AHI ≥ 30) [18,19]. The simple snorers according to PSG results (AHI was <5/h) were excluded from the study.

Body mass index (BMI) was another investigated criteria. Patients were divided into three groups: BMI < 25 as normal weight, BMI from 25 to 30 as overweight, and BMI > 30 as obese patients.

The last parameter investigated was the duration (days) of hospitalization. Patients were divided into 3 groups: <2 days of hospitalization, from 3 to 6 days, and over 6 days.

The average time interval between surgery and the interview was 26 months, with a range from 2 to 56 months.

### 2.3. Investigated Parameters

Swallowing problems, foreign body sensation in the throat, painful sensation, and nose regurgitation evaluated in the postoperative time were initially reported. Subjective outcomes in a long-term follow-up using the PPOPS Questionnaire were also evaluated and analyzed [17]. All the patients were interviewed and completed the PPOPS questionnaire, which is detailed in Table 1. This consists of 12 questions, and the answers recorded scored from 0 to 3 with a total maximum score of 36.

The first three questions of the PPOPS investigate swallowing problems and their resolution after surgery. Questions 4 and 5 investigate problems related to velopharyngeal insufficiency, such as regurgitation of liquid in the nose and rhinolalia. The next three questions regard loss of weight, foreign body sensation in the throat, and the perception of sticky mucus in the throat. Pain after surgery while swallowing and at rest are investigated in questions 9 and 10. The next question asks patients about any difference or worsening of throat sensations at the time of the interview. The last question deals with patients’ general perception of surgery; in fact, it asks them if they would encourage other patients to undergo BRP surgery or not.

The PPOPS was submitted to patients by telephone, and the average duration of the interview was 5 min. One author, well trained on the questionnaire data, dealt with interviewing the patients in order to obtain as much detailed information as possible.

### 2.4. Statistical Analysis

A descriptive analysis of data is reported. The Chi-square test was used for correlating OSA severity and postoperative outcomes. The Wilcoxon signed-rank test was used to compare preoperative and postoperative results of AHI. A *p* value of <0.05 was taken as the threshold of statistical significance.

This research study was performed in accordance with the principles of the Declaration of Helsinki and approved by the local Ethics Committee (Ethic Project Identification Code: RIF. CE 4842 20-06-2019). Informed consent was obtained from all individual participants included in the study.

## 3. Results

The characteristics of the enrolled patients are reported in Table 2.

### 3.1. PPOPS Results

The average score of the PPOPS in the study group was 9.57 with a range between 0 and 24.

**Figure 1:**
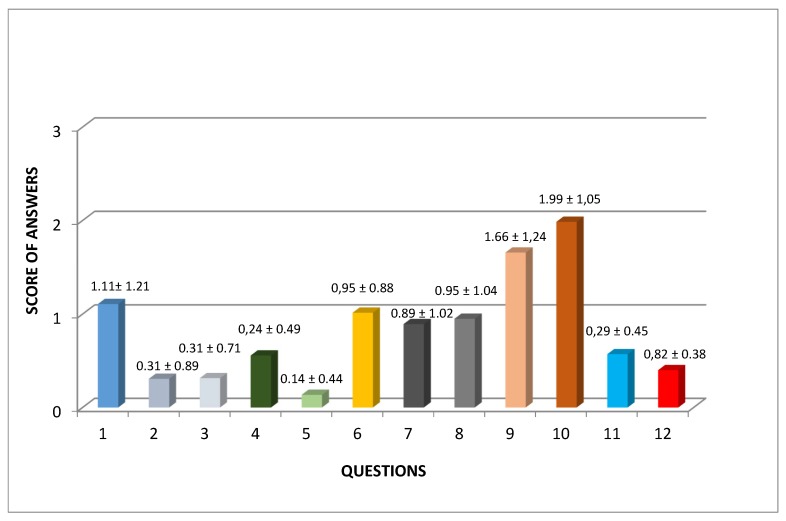
Average scores for each single question of the PPOPS and standard deviation for single questions are reported on each column of the table.

In Figure 1, the average scores for each single question (value 3 as the worst possible result) are shown. The worst results were obtained for questions 9 (Score 1.66) and 10 (score 1.99), which were the questions that investigated postoperative pain.

### 3.2. Swallowing Problems

Forty-nine percent of patients did not complain of swallowing problems after surgery, 11% reported problems for few days, 21% had problems for some weeks, and 19% of patients had problems in the first month after surgery.

At the time of the PPOS interview, 91% of patients did not complain of residual swallowing problems, while in the remaining 9% of patients, these persisted and consisted of mild dysphagia in 8% and moderate dysphagia in 1%. No patients complained of severe residual dysphagia.

**Figure 2:**
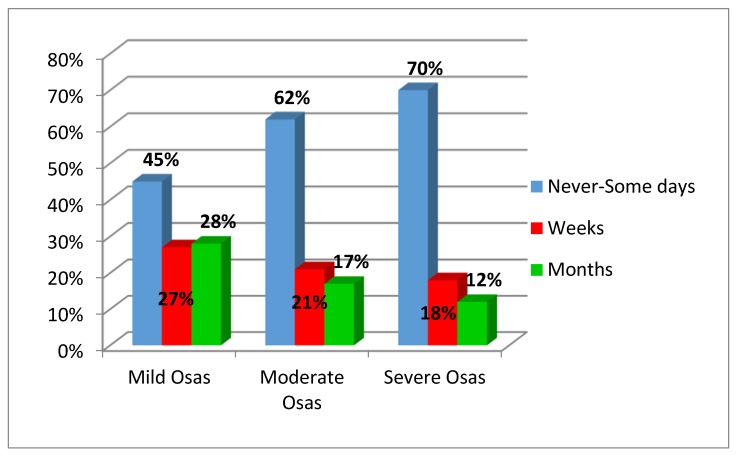
Relation between obstructive sleep apnea syndrome severity and swallowing problems.

Swallowing problems were inversely correlated with OSA severity with a statistical significance (Figure 2, *p* = 0.04).

Seventy percent of the patients aged <40 years had no or only mild swallowing difficulties after surgery. On the other hand, in the other age groups (41–64 and >65 years), the number of patients with mild swallowing difficulties or no difficulty was 58% and 54%, respectively. Hence, older patients had greater swallowing difficulties after surgery. However, swallowing problems did not show any statistical difference in the age groups studied (*p* > 0.1).

### 3.3. Rhinolalia

Rhinolalia was present in 8% of patients at the time of POPPS investigation, mild in 6% of cases and moderate in 2% of cases. The remaining 92% of patients did not describe this problem. No correlation between age groups or OSA severity with rhinolalia problems emerged (*p* > 0.05 in both analysis).

### 3.4. Nose Regurgitation

This complaint was described in only 11% of cases after surgery. Of these patients, 2% presented this problem at the time of evaluation (1% rarely and 1% often). The remaining 98% did not have this problem at the time of follow-up.

### 3.5. Loss of Weight

In 64% of cases, a weight loss from 0 to 5 kilos was reported after surgery, while 36% of patients lost from 6 to 20 kilos. 

### 3.6. Foreign Body Sensation in the Throat

Among all patients, 52% complained of a foreign body sensation in the throat in the days after surgery, in 24% of these cases the problem was of mild intensity, in 19% it was moderate, and in only 9% of cases it was severe.

In 80% of these patients the problem resolved over the months. In 20% of patients the foreign body sensation was still present at the time PPOPS evaluation.

The relationship between age and foreign body sensation had no statistical relevance (*p* = 0.2). Similarly, no statistical difference between foreign body sensation and OSA severity emerged (*p* = 0.3).

### 3.7. Sticky Mucus in the Throat

Fifteen percent of patients experienced a sensation of sticky mucus in throat at the time of evaluation. This sensation was related to the BMI of the group of study and proved to be statistically significant with a *p*-value of 0.02. 

### 3.8. Painful Sensation

Painful sensation in the throat immediately after surgery was found to be a very common problem in all patients. Throat pain at rest was reported by 44% of patients. Throat pain after surgery during swallowing was described by 71% of patients. Pain was mild in 11% of cases, moderate and severe in 24% and 36% of cases, respectively.

Throat pain decreased over time. In fact, 100% of the patients enrolled in the study reported that they had no pain in the throat during swallowing at the time of the PPOPS interview. Obviously, an inverse relation emerged between postoperative painful sensation and the time interval from surgery (regression analysis *p* = 0.001).

### 3.9. Discourage Others from Undergoing the Procedure?

Only 16% of patients said they would discourage other patients from undergoing the procedure, 7% of patients did not have an answer to this question, and lastly, 77% would not discourage others but would rather advise the procedure.

Table 3 summarized all subjective outcomes in the short-term evaluation and long-term follow-up.

### 3.10. Preoperative and Postoperative AHI

In the enrolled patients, the mean preoperative value of AHI was 31.5 (Standard Deviation = 17). After BRP surgery (last follow-up evaluation), a mean value of AHI of 11.4 (standard deviation = 10.3) was calculated. There was a statistical difference between preoperative and postoperative AHI (*p* = 0.00001; Z = −9.7).

## 4. Discussion

UPPP and other previous palatal surgery techniques (e.g., LAUP, UPF) for OSA were based on ablative techniques to remove the uvula and a significant amount of soft palate tissue. Over the years, these procedures were associated with a high incidence of unfavorable postoperative complications and morbidities [13,14,15,16]. In the short and long term, the most typical problems reported in these patients were dysphagia, rhinolalia, velopharyngeal insufficiency and nasopharyngeal regurgitation, phlegm in throat, and abnormal scarring with velopharyngeal stenosis [13,14,15,16]. Besides, the tissue resection required for these techniques caused a thick fibrotic scar on the palatal edge that, touching and abrading the base of the tongue, resulted in throat discomfort or foreign body sensation in the throat [17,20,21].

In recent years, lateral pharyngeal wall collapse has been reconsidered in the OSA pathogenesis, and palatal surgical techniques have evolved from ablative techniques towards the remodeling of the pharyngeal lateral walls and enlargement of the velopharyngeal lumen [7,8].

Different authors agree that the more recent palatal surgery techniques (e.g., ESP, BRP), based on more reconstructive principles that respect the lateral pharyngeal walls and preserve some or part of the uvula, appear to have less long-term postoperative complications and morbidities [7,8,9]. Pang et al. [12], analyzing the long-term complications of palatal surgery in different surgical techniques, reported that the procedures with the highest incidence of long-term complaints were the UPPP, uvulopalatal flap, and relocation pharyngoplasties, whereas the lowest symptom rate was described by patients who underwent ESP or BRP. The number of complication complaints (defined as a complaint of any one of the above symptoms) per procedure was as follows: (1) UPPP, 64 procedures, 32 symptom complaints; (2) ESP, 50 procedures, zero symptom complaints; (3) BRP, 40 procedures, seven symptom complaints; (4) UPF, 11 procedures, five symptom complaints; (5) RP, eight procedures, eight symptom complaints.

In the study reported by Pang et al. [12], 50 patients who underwent ESP did not complain of any unwanted postoperative symptoms or have complaints at long-term follow-up. On the other hand, in 40 BRP procedures, seven patients presented complaints several months after surgery (two cases of dry throat, one case of throat lump, and four cases of throat phlegm).

An important aspect that should be considered is that many of the postoperative complications of palatal surgery are related to the patient’s subjective perceptions. Therefore, for an accurate evaluation of outcomes after surgery, it is necessary to explore the patients’ subjective perception [22].

In 2018, in order to investigate the subjective outcome of palatal surgery, Rashwan et al. [17] created a questionnaire, the Palate Postoperative Problem Score (PPOPS). This questionnaire investigated patients’ perception and the most common problems arising after surgery in the long and short term.

The PPOPS questionnaire is today the only questionnaire published in the literature that analyzes the postoperative outcomes and patients’ feedback following palatal surgery procedures. It is able to provide us with a clear assessment of the Quality of Life (QOL) related to each one of the most common complaints described by patients who underwent palatal surgery. Understanding all the possible postoperative complications and subjective outcomes of palatal surgery is essential before choosing the type of surgical technique. This validated questionnaire employs a result scale with intermediate values, so that it truly reflects patients’ responses because it is scale that goes from the worst possible result to the best one, passing through partially positive and/or partially negative results [23].

In their original report, Rashwan et al. [17] used PPOPS to evaluate the most common problems arising after surgery in BRP and ESP techniques. The BRP group value of questionnaire was 4.05, while the questionnaire value of ESP group was 4.35. Modica et al. [24] used the same questionnaire to evaluate the results of different palatal surgery techniques. They reported a value of 5.92 for the ESP, 2.8 for the UPPP, and 2.4 for the BRP technique.

In this paper, we have analyzed the long-term subjective outcomes of a large group of patients undergoing barbed reposition pharyngoplasty using the PPOPS questionnaire.

The observation period of the study ranged from 2 months to 56 months with an average follow-up of 26 months.

The total average PPOPS score of the patients enrolled in the study was 9.57, higher than the average score obtained in the initial study of Rashwan et al. and in the study of Modica et al. [24] that reported an average score of 4.05 and 2.4 for the BRP technique. This worst result could be related to the high number of cases enrolled in the present study and to the longer time of observation. However, considering the worst possible score of PPOPS, namely 36, the average score of 9.57 obtained can be considered indicative of a good subjective general outcome after surgery with a lower incidence of complication.

The results obtained from the study show that patients complain of swallowing problems during the first weeks and first month of surgery (21% and 19% of patients), probably due to the scarring and reabsorption of the barbed suture in the palate tissue. At long-term follow-up, only 8% of patients had mild dysphagia and 1% moderate dysphagia. No patients complained of severe residual dysphagia. These results confirmed the findings of Montevecchi et al. [10], who studied the effect of BRP on swallowing using the Anderson Dysphagia Inventory (MDADI) questionnaire. The authors reported a preoperative mean MDADI score of 3.67 ± 2.58, whilst the postoperative first week and 1 month scores were 11.18 ± 4.32 and 5.06 ± 1.83, respectively [10].

Since rhinolalia and nasal regurgitation due to velopharyngeal insufficiency were reported in a very low percentage of cases of enrolled patients (8% and 2%, respectively), we can consider these minor BRP-related complications.

The complications/morbidity of BRB surgery for OSAS would seem to be inferior to those reported by other authors for ablative techniques such as the classical UPPP.

Regarding postoperative complications, Goh et al. [25] reported velopharyngeal incompetence in 28.5% of patients who underwent UPPP. Tang et al. [26] themselves, in a systemic review of 24 studies dealing with classic UPPP, found a lower incidence of velopharyngeal insufficiency (VPI) (8.1%), but swallowing difficulty in 17.7%, dry pharynx in 23.4%, voice changes in 9.5%, and taste disturbances in 8.2% of patients. Besides, foreign body sensation/lump in the throat was described by 31.2% of patients. In the study of Värendh et al. [27], 49/144 patients (38%) who underwent UPPP reported problems, consisting of nasal regurgitation in 14%, swallowing in 20%, voice changes in 12%, and pain in the oral cavity in 12% of cases.

Foreign body sensation in the throat was reported by 52% of patients in the period immediately after surgery, but it was reported by only 20% of patients at the time of the PPOPS interview.

Sticky mucus in the throat was described by 15% of the patients enrolled in the present study. This sensation was related to BMI and was probably due to a laryngopharyngeal reflux, which affects most OSA patients with a high BMI [20].

Throat pain is certainly a relevant issue in BRP surgery and should always be considered and communicated to patients before submitting them to BRP. Pain during swallowing was described by 71% of enrolled patients. Nevertheless, in these cases, a reverse correlation between throat pain during swallowing and the amount of time which had elapsed from surgery emerged.

Finally, a point not to be underestimated is the positive impression that patients have had from this type of surgery. In fact, the majority of such patients would encourage other patients to undergo BRP surgery.

### Strengths and Limitations

In our opinion, the strength of this study lies in the large number of patients examined and the long period of observation with an average follow-up of 26 months. Besides, a validated questionnaire [17,23] was used to investigate patients’ subjective outcomes.

A limitation of the study could be that the BRP technique has not been compared with other palatal surgical techniques for OSA in order to find differences in subjective outcomes. Further studies are under way to evaluate this aspect.

## 5. Conclusions

PPOPS has been helpful in assessing the subjective evaluation of an innovative and new palatal surgery, namely barbed reposition pharyngoplasty. Using this specific questionnaire, it has been demonstrated that the BRP technique provides efficacy and a lower morbidity, thus ensuring an excellent subjective and functional result with few long-term complications after surgery.

The large number of cases enrolled in the study group and the long period of time taken into consideration in the study are aspects that endorse our results.

## Figures and Tables

**Table 1:** Palate Postoperative Problem Score (PPOPS) questionnaire.

Name____________________________________		Age____		
**Date and type of operation**______________________________________________ **Date of questionnaire and examination**____________________________________				
***QUESTIONS***	***SCORE***			
	***0***	***1***	***2***	***3***
(1) Did you have any swallowing problems after surgery	Never	Days	Weeks	Months
(2) The problems resolved	Spontaneously	With a doctor	With the speechterapist	Unresolved
(3) Residual difficulty in swallowing	No	Mild	Moderate	Severe
(4) Residual nasal tone of voice	No	Mild	Moderate	Severe
(5) Residual regurgitation of liquid into the nose	Never	Rarely	Often	Always
(6) Any weight loss recorded after surgery	No	Mild	Moderate	Severe
(7) Any foreign body sensation in the throat	No	Mild	Moderate	Severe
(8) Do you feel sticky mucus in the throat	No	Mild	Moderate	Severe
(9) Painful sensation in the throat at rest	No	Mild	Moderate	Severe
(10) Painful sensation in the throat while swallowing	No	Mild	Moderate	Severe
(11) Do you have different ad a worse feeling in the throat after surgery	No	Mild	Moderate	Severe
(12) Do you discourage the procedure to others	No	Mild	Moderate	Severe
	**Total Score**_______			
Item for each score from 0 to 3				
**Total Score 0/36**				

**Table 2:** Enrolled patients’ characteristics.

	Number of Patients	Percentage
**Sex**		
Female	10	7%
Male	130	93%
**Age**		
<40	33	24%
40–65	94	67%
>65	13	9%
**OSA severity**		
Mild	53	38%
Moderate	29	21%
Severe	49	35%
**BMI**		
<25	31	26%
25–30	62	51%
>30	28	23%
**Type of surgery**		
BRP and nasal surgery	130	93%
Only BRP surgery	10	7%
**Days of hospitalization**		
<2	16	11%
3–6	103	74%
>6	21	15%

Obstructive sleep apnea (OSA), Body mass index (BMI), barbed reposition pharyngoplasty (BRP).

**Table 3:** Subjective outcomes in the short-term evaluation and long-term follow-up.

ITEM	Never N/%	Days N/%	Weeks N/%	Months N/%
**Swallowing problems in the postoperative period**	69/49	15/11	29/21	27/19
	**No** **N/%**	**Mild** **N/%**	**Moderate** **N/%**	**Severe** **N/%**
**Residual Swallowing difficult** (PPOPS evaluation)	127/91	11/8	2/1	0/0
**Rhinolalia** (PPOPS evaluation)	129/92	8/6	3/2	0/0
	**Never** **N/%**	**Rarely** **N/%**	**Often** **N/%**	**Always** **N/%**
**Nose regurgitation** (PPOPS evaluation)	138/98	1/1	1/1	0/0
	**0–5 Kg** **N/%**		**6–20 Kg** **N/%**	
**Weight loss** (PPOPS evaluation)	90/64		50/36	
	**No** **N/%**	**Mild** **N/%**	**Moderate** **N/%**	**Severe** **N/%**
**Foreign body in throat after surgery**	67/48	34/24	27/19	12/9
**Sticky mucus in throat**	119/85	18/13	3/2	0/0
**Pain in throat at rest after surgery**	92/66	14/10	20/14	28/20
**Pain in throat during swallowing after surgery**	41/29	15/11	34/24	50/36
	**YES** **N/%**		**NO** **N/%**	**I don’t know** **N/%**
**Discourage others to procedure?** (PPOPS evaluation)	22/16		108/77	10/7
